# First record of *Onchidium reevesii* from Korean tidal flats (Mollusca, Gastropoda, Onchidiidae)

**DOI:** 10.3897/BDJ.14.e189286

**Published:** 2026-06-18

**Authors:** Bongjun Kim, Inho Yang, Sang-kyu Lee, Hyesuk Kim, Jong Seong Khim, Jongseong Ryu, Jinsoon Park

**Affiliations:** 1 Department of Marine Bioscience and Environment, National Korea Maritime & Ocean University, Busan, Republic of Korea Department of Marine Bioscience and Environment, National Korea Maritime & Ocean University Busan Republic of Korea; 2 Department of Convergence Study on the Ocean Science and Technology, National Korea Maritime & Ocean University, Busan, Republic of Korea Department of Convergence Study on the Ocean Science and Technology, National Korea Maritime & Ocean University Busan Republic of Korea; 3 School of Earth and Environmental Sciences and Research Institute of Oceanography, Seoul National University, Seoul, Republic of Korea School of Earth and Environmental Sciences and Research Institute of Oceanography, Seoul National University Seoul Republic of Korea; 4 Research Institute of Ocean Science and Technology, National Korea Maritime & Ocean University, Busan, Republic of Korea Research Institute of Ocean Science and Technology, National Korea Maritime & Ocean University Busan Republic of Korea; 5 School of Future Convergence (II), Seo Kyeong University, Seoul, Republic of Korea School of Future Convergence (II), Seo Kyeong University Seoul Republic of Korea

**Keywords:** DNA barcoding, integrative taxonomy, Korean tidal flats, Onchidiidae, *Onchidium reevesii*

## Abstract

**Background:**

Air-breathing marine slugs of the family Onchidiidae inhabit intertidal environments worldwide, but their diversity and distribution remain poorly documented in Korean tidal flats, particularly in upper intertidal and supralittoral habitats such as saltmarsh vegetation.

**New information:**

The following report documents the first confirmed record of *Onchidium reevesii* from Korea, based on specimens collected from saltmarsh vegetation in the upper intertidal and supralittoral zones of Korean tidal flats at three localities: Jindo, Muan and Suncheon. Species identification was facilitated by an integrative taxonomic approach, incorporating external morphology, radular characters and mitochondrial COI and 16S rRNA sequence analyses. The Korean specimens closely matched the diagnostic morphological features described for Chinese specimens in previous studies. Furthermore, molecular analyses revealed a high degree of sequence similarity, which supports a close phylogenetic affinity with reported sequences of *O. reevesii*. This record constitutes the first genetic confirmation of *O. reevesii* on the Korean Peninsula, thereby extending the known geographic distribution of the species across the Yellow Sea in East Asia.

## Introduction

The western and southern coasts of Korea play host to extensive tidal flats, which represent one of the most ecologically significant coastal ecosystems in the Yellow Sea region (Koh and Khim 2014). These habitats support highly diverse benthic communities (Park et al. 2014). Recent integrative taxonomic studies in Korea have revealed previously unrecorded species across various taxa (Do et al. 2020, Cho et al. 2023, Lee et al. 2025). These findings suggest that tidal-flat ecosystems may also harbour substantial undocumented biodiversity, including non-native and cryptic species, highlighting the need for further taxonomic investigation.

The family Onchidiidae consists of air-breathing sea slugs that inhabit intertidal environments across much of the world. Those shell-less gastropods have particularly high species richness in the coastal ecosystems of Southeast Asia (Goulding et al. 2018). However, species boundaries and nomenclatural assignments within Onchidiidae have long been difficult to resolve using morphology alone, resulting in persistent taxonomic ambiguity at the species level (Dayrat 2009). To address this issue, recent studies have adopted integrative taxonomic approaches that combine morphological and molecular data. As a result, species delimitation has become more robust and evolutionary relationships within the family have been clarified (Dayrat et al. 2016, Dayrat et al. 2017, Goulding et al. 2018, Dayrat et al. 2019a, Dayrat et al. 2019b, Goulding et al. 2022).

In contrast to the global diversity of onchidiid slugs, records from Korea are extremely limited. The only record of onchidiid slugs from Korean tidal flats, reported from Muan in south-western Korea, was identified as *Onchidium hongkongensis*. However, identification was based exclusively on photographic evidence (Kil and Lee 2011). The species reported from Korea as *Onchidium hongkongensis* likely represents a misspelling of *Onchidium hongkongense*, as the former name is not recognised as a valid species in the World Register of Marine Species. Subsequent taxonomic revisions have treated *Onchidium hongkongense* as a junior synonym of *Paromoionchis tumidus* (Dayrat et al. 2019a). Consistently, in the National Institute of Biological Resources database, *Onchidium hongkongensis* is not recognised as a valid species and *Onchidium hongkongense* is treated as a synonym of *Paromoionchis tumidus* (NIBR 2025). Accordingly, the taxonomic status of the genus *Onchidium* in Korean coastal habitats remains uncertain.

In this study, we report the first confirmed presence of *Onchidium reevesii* in the upper intertidal and supralittoral zones of tidal flats in Jindo, Muan and Suncheon in Korea. Species identification was supported by an integrative approach combining detailed morphological observations with analyses of the mitochondrial COI and 16S genes. This finding is notable, as *O. reevesii* was previously known only from China, with its type locality also being located within the region (Dayrat et al. 2016).

Thus, the current study expands the known geographic distribution of *O. reevesii* and offers new insights into the biogeography of the genus *Onchidium* in East Asia, contributing to a more accurate understanding of onchidiid diversity in Korea.

## Materials and methods

**Sample preparation**

**Figure 1.**
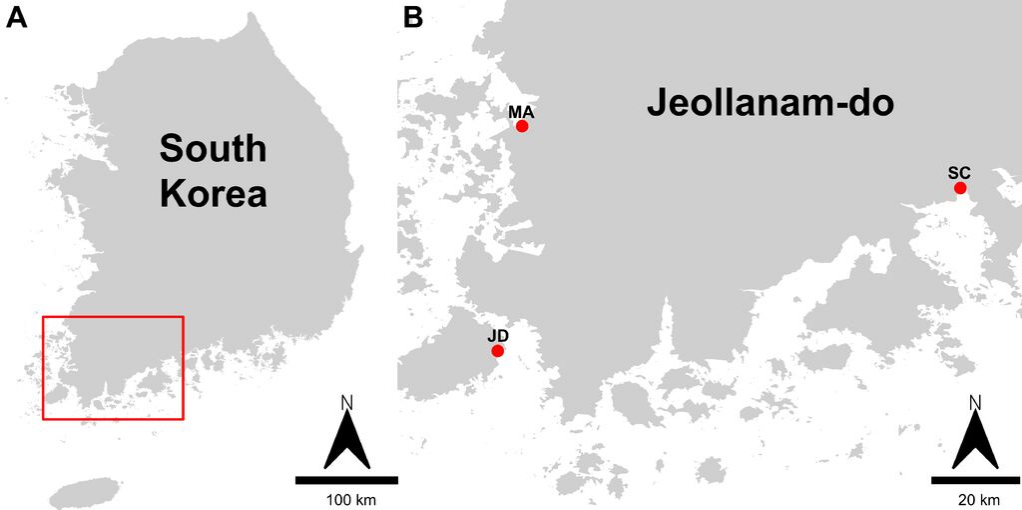
Sampling sites of *Onchidium reevesii* in Korea. **(A)** Map of South Korea, showing the study region (red rectangle); **(B)** Enlarged map of Jeollanam-do, with the three sampling sites: JD (Jindo), MA (Muan), SC (Suncheon).

**Figure 2.**
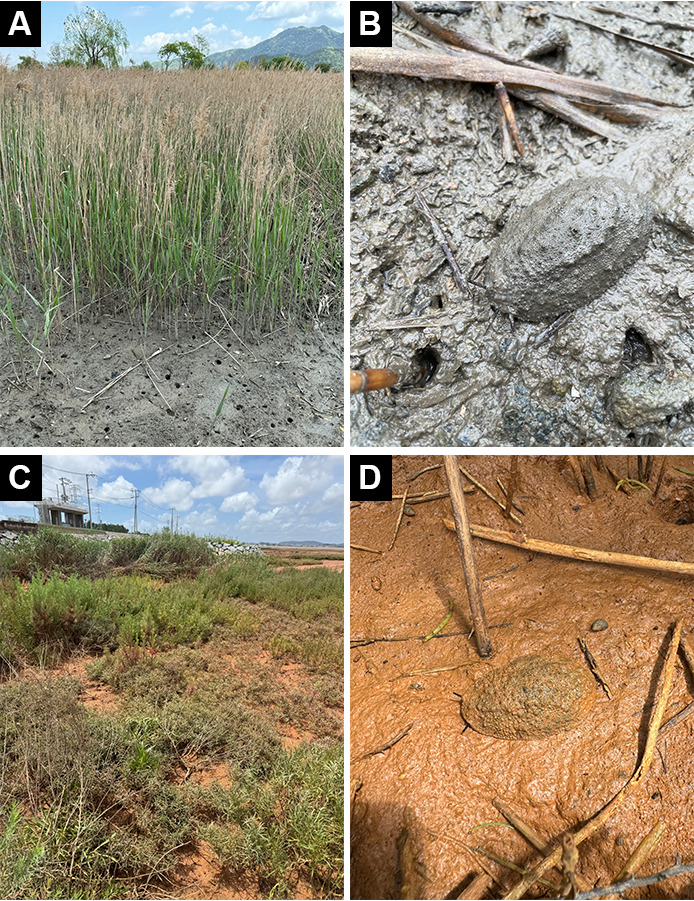
Habitat characteristics and live individuals of *Onchidium reevesii* in Korea. **A** Upper intertidal to supralittoral saltmarsh habitat at the Suncheon locality (SC); **B** Live individual of *O. reevesii* on wet muddy substrate beneath saltmarsh vegetation at Suncheon (SC); **C** Upper intertidal to supralittoral saltmarsh habitat at the Muan locality (MA); **D** Live individual of *O. reevesii* on wet muddy substrate beneath saltmarsh vegetation at Muan (MA).

Specimens of *Onchidium reevesii* were collected from three localities along the southern and western coasts of Korea: Jindo, Muan and Suncheon (Fig. 1). A total of two individuals were collected from Jindo on 11 July 2024, four individuals from Suncheon on 23 August 2024 and three individuals from Muan on 13 August 2025. All specimens were obtained from saltmarsh vegetation within the upper intertidal and supralittoral zones during periods of maximum low tide. Habitat conditions were recorded through in situ photography at the time of collection (Fig. 2) and additional images of live specimens were obtained in the laboratory prior to preservation. Before photographing in the laboratory, specimens were gently rinsed with distilled water using a squeeze bottle to remove surface mud and debris. All specimens were fixed in 95% ethanol for subsequent morphological and molecular analyses. All maps presented in this study were generated using QGIS version 3.40.13 (QGIS Development Team).

Muscle tissue from the specimens was used for DNA extraction. Tissue samples were obtained from all individuals collected from Jindo, Suncheon and Muan. The resulting sequence data were deposited in the NCBI database and made available through GenBank. Sequence submissions were handled using the BankIt submission portal, which performs automated checks on file format and sequence quality. Submission data included information on taxonomy, genetic markers, sampling locality, geographic coordinates, collection dates and the names of collectors and identifiers. Associated trace files provided information on primer sets, sequence orientation and the genetic markers employed. COI and 16S sequences were edited, aligned in FASTA format, assigned individual sample IDs and uploaded to GenBank. Photographs of specimens and field habitats were taken using an iPhone 15 Pro camera. GenBank accession numbers for all sequences generated in this study are provided (Table 1).

For scanning electron microscopy (SEM), a portion of the buccal mass from specimen MA2, collected in Muan, was dissected and immersed in a 10% sodium hydroxide (NaOH) solution for seven days; the solution was refreshed at two-day intervals. Following complete dissolution of the surrounding tissues, the radula was isolated, rinsed thoroughly with distilled water and cleaned using three successive ultrasonic treatments of one minute each. The cleaned radula was then mounted on aluminium stubs using carbon tape, air-dried at room temperature for approximately one hour and subsequently sputter-coated with gold. SEM imaging was carried out using a CLARA GM field-emission scanning electron microscope (Tescan, Brno, Czechia).

### Primer sequences

Genomic DNA was extracted from approximately 2 mg of muscle tissue using the HiGene Genomic DNA Prep Kit (Biofact, Daejeon, Korea). Two mitochondrial gene regions, COI and 16S rRNA, were amplified for sequencing. The COI fragment was amplified using the primers LCO1490 (5′-GGTCAACAAATCATAAAGATATTG-3′) and HCO2198 (5′-TAAACTTCAGGGTGACCAAAAAATCA-3′) (Folmer et al. 1994). The 16S rRNA region was amplified using the primers 16Sar (5′-CGCCTGTTTATCAAAAACAT-3′) and 16Sbr (5′-CCGGTCTGAACTCAGATCACGT-3′) (Palumbi 1996). PCR products from both COI and 16S were sequenced in both directions using forward and reverse primers.

### Target PCR mixture

Each PCR amplification (COI and 16S) was performed in a total reaction volume of 30 μl. The reaction mixture consisted of 15 μl of BioFACT Lamp Taq Master Mix 2, 1 μl of forward primer (10 pmol/μl), 1 μl of reverse primer (10 pmol/μl), 2 μl of genomic DNA template and 12 μl of distilled water.

### PCR condition and sequencing

The thermocycling protocol involved separate PCR procedures for COI and 16S. For COI, the PCR programme consisted of an initial denaturation at 95°C for 15 minutes, followed by 40 cycles of denaturation at 95°C for 20 seconds, annealing at 50°C for 40 seconds and extension at 72°C for 1 minute, with a final extension at 72°C for 5 minutes. The 16S PCR was performed under the same conditions, except that the annealing temperature was set to 54°C.

Amplified PCR products were confirmed by electrophoresis on a 1% agarose gel and visualised under UV illumination after staining with EcoDye DNA Staining Solution (Biofact). Sequencing reactions were performed using the BigDye Terminator v.3.1 Cycle Sequencing Kit (ABI, Waltham, USA) and all PCR products were sequenced on a 3730XL DNA Analyzer (ABI) at the Biofact sequencing facility.

### Phylogenetic analyses

Phylogenetic relationships were assessed separately for the COI and 16S rRNA datasets. For the COI analysis, we used a total of 13 sequences of *Onchidium reevesii*, including eight sequences newly generated in this study and five sequences available in GenBank. To provide a broader taxonomic framework within the genus *Onchidium*, two additional congeners (*O. stuxbergi* and *O. typhae*) were incorporated, represented by six sequences in total. *Paromoionchis tumidus* was selected as the outgroup. For the 16S analysis, we used 14 sequences of *O. reevesii* in total, comprising nine sequences produced in this study and five additional sequences obtained from GenBank. The same two *Onchidium* congeners and the identical outgroup taxon were included as in the COI dataset. Sequence alignments were performed using the ClustalW algorithm (Thompson et al. 1994), as implemented in MEGA 12 (Kumar et al. 2024). Maximum Likelihood (ML) phylogenetic analyses were conducted using IQ-TREE v.3.0.1 (Wong et al. 2025). The best-fit nucleotide substitution models were selected using ModelFinder (Kalyaanamoorthy et al. 2017), implemented in IQ-TREE, based on the Bayesian Information Criterion (BIC). The selected models were HKY+F+G4 for the COI dataset and TPM3u+G4 for the 16S rRNA dataset. Branch support was assessed using the ultrafast bootstrap approximation (UFBoot) with 1,000 replicates (Hoang et al. 2017). Bootstrap values greater than 65% were considered to indicate strong nodal support. Analytical settings were kept consistent between the COI and 16S datasets. The resulting phylogenetic trees were visualised and edited using MEGA 12.

## Taxon treatments

### Onchidium reevesii

(Gray, 1850)

35F78ABD-F51A-5495-8DC2-C1315E150A2F

https://www.marinespecies.org/aphia.php?p=taxdetails&id=956738

Onchidella reevesii Gray, 1850
Onchidium reevesii (Gray, 1850): Dayrat et al. (2016).
Onchidium struma (nomen nudum): The name *Onchidium struma* has been widely used in the Chinese literature since the early 1990s and its biology, physiology, biochemistry and husbandry have been studied. However, the species has never been formally described.
Paraoncidium reevesii (Gray, 1850).

#### Materials

- **Type status:** Other material. **Occurrence:** catalogNumber: JD1; recordedBy: Sang-kyu Lee; occurrenceID: E769BD7A-E3FB-5979-9A44-DC5C95897BC3; **Taxon:** scientificName: *Onchidium reevesii*; **Location:** locality: Gogun-myeon, Jindo-gun, Jeollanam-do, Korea; decimalLatitude: 34.4809; decimalLongitude: 126.3585; **Event:** eventDate: 11-07-2024
- **Type status:** Other material. **Occurrence:** catalogNumber: JD2; recordedBy: Sang-kyu Lee; occurrenceID: 844DF95E-A579-50A2-98E7-B0F1B8CC8540; **Taxon:** scientificName: *Onchidium reevesii*; **Location:** locality: Gogun-myeon, Jindo-gun, Jeollanam-do, Korea; decimalLatitude: 34.4809; decimalLongitude: 126.3585; **Event:** eventDate: 11-07-2024
- **Type status:** Other material. **Occurrence:** catalogNumber: MA1; recordedBy: Bongjun Kim; occurrenceID: C02BF28D-B275-5D36-BD90-A574A83C0CDA; **Taxon:** scientificName: *Onchidium reevesii*; **Location:** locality: Hyeongyeong-myeon, Muan-gun, Jeollanam-do, Korea; decimalLatitude: 35.0275; decimalLongitude: 126.4265; **Event:** eventDate: 13-08-2025
- **Type status:** Other material. **Occurrence:** catalogNumber: MA2; recordedBy: Bongjun Kim; occurrenceID: 1ABBA389-CC02-50CA-AD81-051D44938A0B; **Taxon:** scientificName: *Onchidium reevesii*; **Location:** locality: Hyeongyeong-myeon, Muan-gun, Jeollanam-do, Korea; decimalLatitude: 35.0275; decimalLongitude: 126.4265; **Event:** eventDate: 13-08-2025
- **Type status:** Other material. **Occurrence:** catalogNumber: MA3; recordedBy: Bongjun Kim; occurrenceID: 050EBFD1-63FF-53BC-BF0E-AF8D55D8F91B; **Taxon:** scientificName: *Onchidium reevesii*; **Location:** locality: Hyeongyeong-myeon, Muan-gun, Jeollanam-do, Korea; decimalLatitude: 35.0275; decimalLongitude: 126.4265; **Event:** eventDate: 13-08-2025
- **Type status:** Other material. **Occurrence:** catalogNumber: SC1; recordedBy: Bongjun Kim; occurrenceID: 63422F18-AF7E-5F4D-A9C5-76A782816AEA; **Taxon:** scientificName: *Onchidium reevesii*; **Location:** locality: Anpung-dong, Suncheon-si, Jeollanam-do, Korea; decimalLatitude: 34.874; decimalLongitude: 127.5004; **Event:** eventDate: 23-08-2024
- **Type status:** Other material. **Occurrence:** catalogNumber: SC2; recordedBy: Bongjun Kim; occurrenceID: 80560472-484C-5ED7-BE95-CFF981772583; **Taxon:** scientificName: *Onchidium reevesii*; **Location:** locality: Anpung-dong, Suncheon-si, Jeollanam-do, Korea; decimalLatitude: 34.874; decimalLongitude: 127.5004; **Event:** eventDate: 23-08-2024
- **Type status:** Other material. **Occurrence:** catalogNumber: SC3; recordedBy: Bongjun Kim; occurrenceID: A2B29FCE-7B5D-51C4-8F5E-06351E45F49A; **Taxon:** scientificName: *Onchidium reevesii*; **Location:** locality: Anpung-dong, Suncheon-si, Jeollanam-do, Korea; decimalLatitude: 34.874; decimalLongitude: 127.5004; **Event:** eventDate: 23-08-2024
- **Type status:** Other material. **Occurrence:** catalogNumber: SC4; recordedBy: Bongjun Kim; occurrenceID: B312E772-0AA8-5EDF-8287-5E2AFE035803; **Taxon:** scientificName: *Onchidium reevesii*; **Location:** locality: Anpung-dong, Suncheon-si, Jeollanam-do, Korea; decimalLatitude: 34.874; decimalLongitude: 127.5004; **Event:** eventDate: 23-08-2024

#### Description

**Figure 3.**
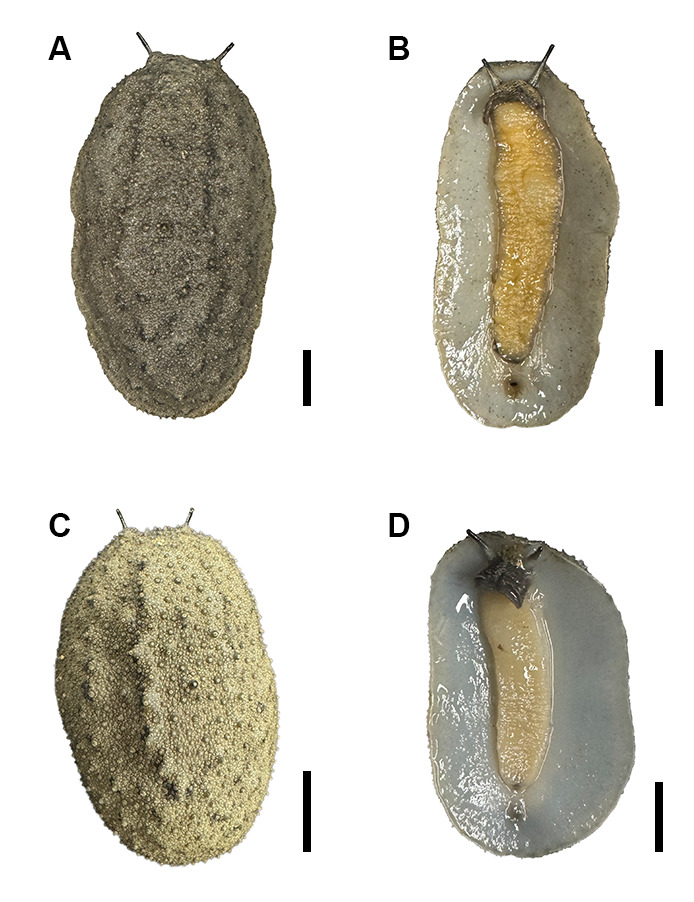
Dorsal and ventral views of live *Onchidium reevesii* specimens from Korea. **A** Dorsal view of SC3 (Suncheon); **B** Ventral view of SC3 (Suncheon); **C** Dorsal view of JD1 (Jindo); **D** Ventral view of SC2 (Suncheon). Scale bar = 1 cm.

**Figure 4.**
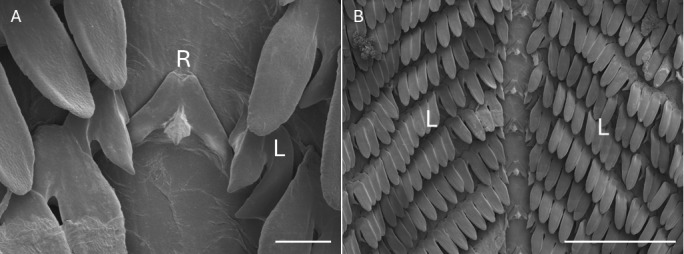
Radula of Korean *Onchidium reevesii*. **A** Rachidian and innermost lateral teeth of MA2 (Muan). Scale bar = 20 μm; **B** Rachidian and lateral teeth of MA2 (Muan). Scale bar = 200 μm. R—rachidian teeth; L—lateral teeth.

Nine specimens were examined. Excluding a juvenile individual (17 mm in body length), the remaining eight living specimens measured 43–74 mm in body length and 27–40 mm in width. The dorsal notum was grey to yellowish-grey, bearing numerous fine papillae distributed evenly across the surface. As reported for Chinese populations of *Onchidium reevesii* (Wang et al. 2018), a distinctly enlarged central papilla (peduncle) was present in the middle of the notum and this feature was clearly observed in the Korean specimens as well. The ventral foot was light yellow to beige, while the mantle surface was light grey with scattered black spots, although some individuals lacked these spots (Fig. 3). Overall external morphology, including colouration and the distribution of papillae, corresponded well with previously documented descriptions of *O. reevesii*. The radular morphology observed in the Korean specimen was consistent with previous descriptions of *Onchidium reevesii* (Wang et al. 2018), including the morphology of the rachidian, innermost lateral and lateral teeth (Fig. 4). Radula observations were based on the Muan specimen (MA2).

#### Molecular data

Partial mitochondrial COI and 16S rRNA sequences were successfully obtained from specimens collected in Jindo, Muan and Suncheon. All sequences were confirmed to be of high quality after manual editing. BLAST searches showed that COI sequences from the Korean specimens exhibited 99.64–99.83% similarity with published *Onchidium reevesii* reference sequences, based on the five closest matches retrieved using specimen SC1, while 16S sequences showed 99.33–99.55% similarity, based on the five closest matches (Table 2). None of the sequences showed BLAST matches to other onchidiid genera, such as *Paromoionchis*, supporting the current morphological identification. All newly-generated sequences have been deposited in GenBank, with accession numbers listed in Table 1. These molecular results represent the first genetic confirmation of *O. reevesii* from Korea and support the presence of this species on the Korean Peninsula.

#### Phylogenetic analyses

Phylogenetic relationships were further assessed using Maximum Likelihood (ML) analyses, based on COI and 16S rRNA sequences. The dataset included eight COI sequences and nine 16S sequences generated from Korean specimens. For BLAST comparisons, the SC1 sample was used to retrieve the five most similar sequences from NCBI BLAST, all of which were confirmed to have been registered from China. To strengthen the robustness of the phylogenetic framework, two additional species of *Onchidium* (*O. stuxbergi* and *O. typhae*) were incorporated and *Paromoionchis tumidus* was included as the outgroup.

**Figure 5.**
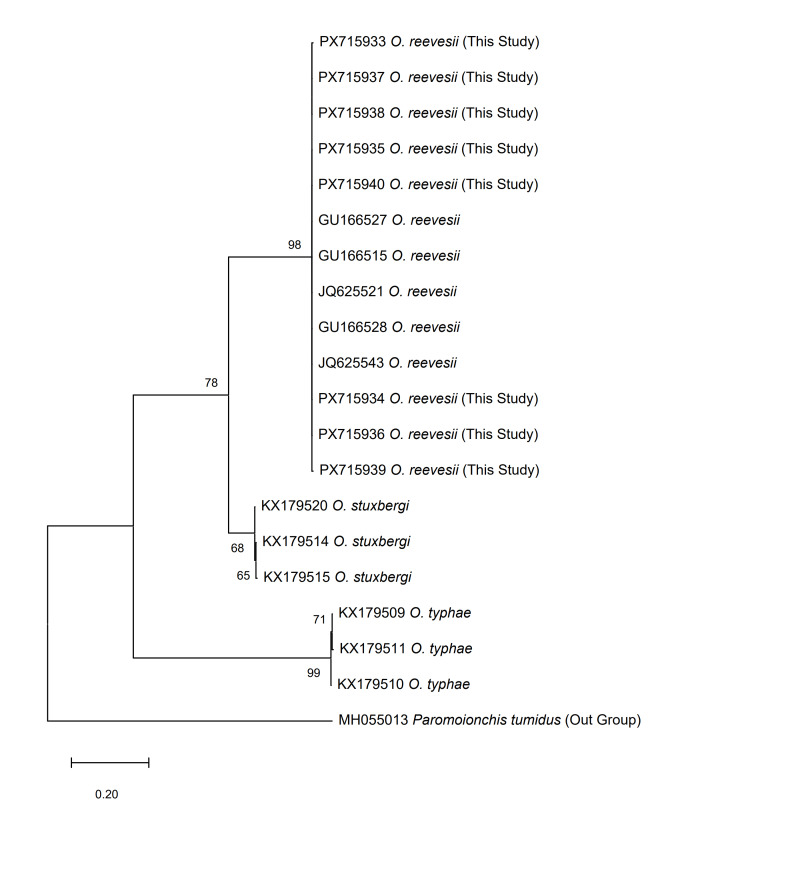
Maximum Likelihood tree of COI sequences of *Onchidium reevesii* collected in Korea. Letters preceding scientific names indicate NCBI accession numbers and sample codes (JD, MA, SC) represent specimens from the three localities in Jeollanam-do. Bootstrap support values are shown at the nodes. Korean specimens cluster with previously published *O. reevesii* sequences, forming a single well-supported clade.

**Figure 6.**
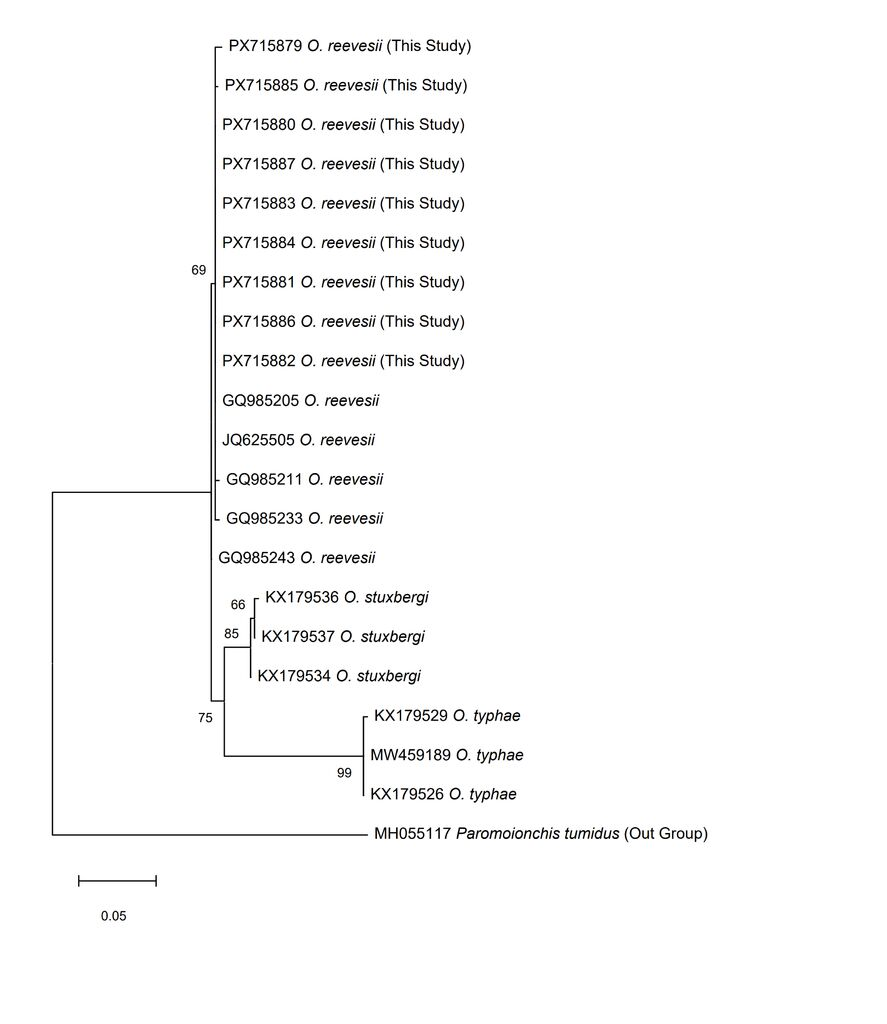
Maximum Likelihood tree of 16S rRNA sequences of *Onchidium reevesii* collected in Korea. Letters preceding scientific names indicate NCBI accession numbers and sample codes (JD, MA, SC) represent specimens analysed in this study. Bootstrap support values are shown at the nodes. Korean specimens form a monophyletic group with previously published *O. reevesii* sequences, consistent with the COI phylogeny.

In the COI Maximum Likelihood (ML) tree, the Korean specimens clustered tightly with Chinese *O. reevesii* sequences, demonstrating clear species-level correspondence. No or only minimal sequence divergence was detected amongst individuals collected from the three Korean localities or between Korean and Chinese populations in the COI dataset (Fig. 5). The 16S ML tree showed a nearly identical topology, further supporting the close genetic affinity between the Korean specimens and *O. reevesii* populations previously reported from China (Fig. 6). The agreement between BLAST identity results and ML phylogenetic patterns provides robust evidence that the Korean specimens represent *O. reevesii*.

#### Ecology and habitat

Specimens were found in the upper intertidal and supralittoral zones of tidal flats, occurring within dense patches of saltmarsh vegetation (Fig. 2). Individuals were observed on moist, muddy substrates that remained saturated during low tide. All specimens were encountered during periods of maximum low tide and none was found in mid- or lower-intertidal areas. The species appears to utilise vegetation cover as a microhabitat, likely benefitting from shaded and humid conditions provided within the saltmarsh environment. All observations and collections were made during summer (July and August), suggesting that the species may be more readily detectable during warmer months, although additional year-round sampling would be needed to confirm seasonal patterns.

#### Distribution

**Figure 7.**
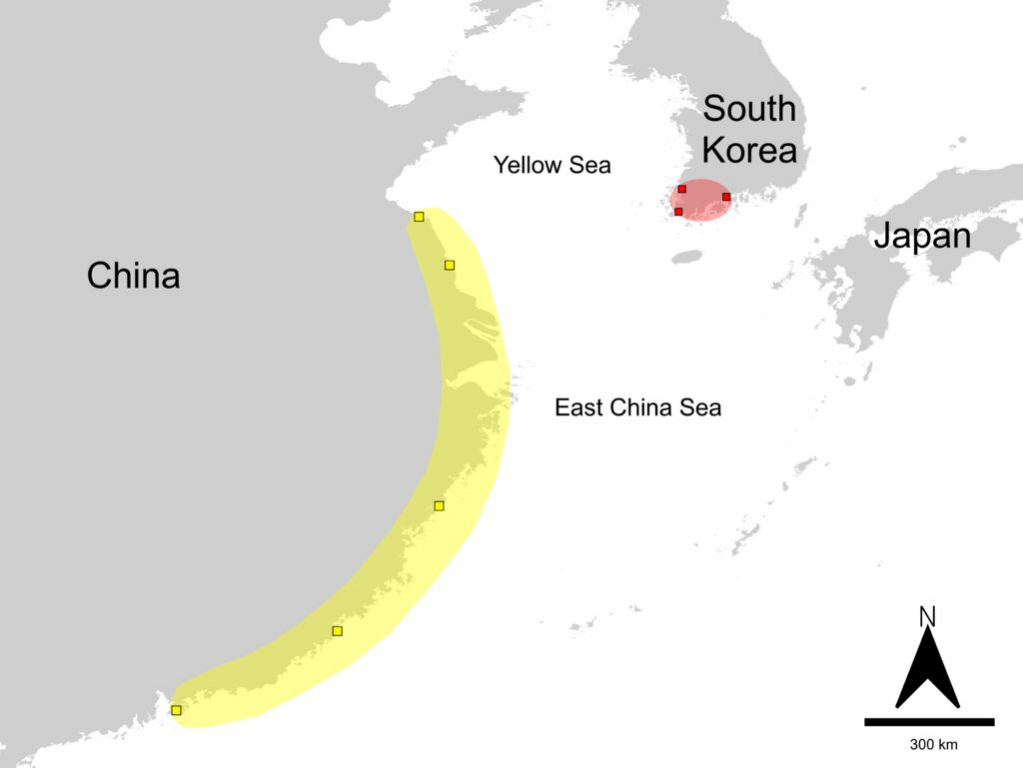
Reported distribution and occurrence of *Onchidium reevesii* (yellow shading and yellow circles), redrawn based on previous studies (Dayrat et al. 2016). Red circles indicate occurrence records from the present study and the red shaded area represents a potential distribution range in Korea, inferred from habitat continuity.

Previously, *Onchidium reevesii* has been recorded only from coastal regions of China, as documented in both the integrative taxonomic revision of the genus (Dayrat et al. 2016) and subsequent regional studies (Wang et al. 2018). The present study provides the first molecularly confirmed record of this species outside China, extending its known distribution range to the Korean Peninsula (Fig. 7).

## Discussion

This study provides the first confirmed record of *Onchidium reevesii* in Korea. The diagnostic morphological traits of the Korean specimens, including notum colouration, the presence of a prominent central dorsal papilla, the distribution of dorsal papillae and radular tooth morphology, closely matched descriptions of Chinese populations. Molecular analyses also supported this identification. The COI and 16S rRNA sequences of Korean specimens clustered with published *O. reevesii* sequences and no intraspecific divergence was detected within the limited dataset examined. While broader sampling will be required to fully assess population-level variation, the currently available evidence strongly supports the identification of the Korean specimens as *O. reevesii*.

To date, only one onchidiid record has been reported from Korean tidal flats, a specimen from Muan identified as *Onchidium hongkongensis* (Kil and Lee 2011). This species has subsequently been treated as a junior synonym of *Paromoionchis tumidus* (Dayrat et al. 2016). The confirmed distribution of *P. tumidus* in the East China Sea and southern Japan (Dayrat et al. 2019a) suggests that its occurrence in nearby Korean tidal flats is possible; however, the earlier Korean record lacks strong evidence and, therefore, precludes definitive evaluation. Moreover, given that the earlier specimen was collected in Muan, the same locality where the present study confirms the presence of *O. reevesii*, the possibility that the previously recorded specimen may have represented *O. reevesii* cannot be ruled out. Accordingly, both the identity of the earlier Korean specimen and the potential presence of *P. tumidus* in Korea merit re-evaluation through additional field collections and concentrated examination.

Before this study, *O. reevesii* was known exclusively from coastal regions of China (Dayrat et al. 2016, Wang et al. 2018). The confirmation of its occurrence in Korea extends the species’ known range across the Yellow Sea to the Korean Peninsula. Together with previous records from China, this finding contributes to a more refined understanding of the regional distribution pattern of the species. The occurrence of this species within saltmarsh vegetation in the upper intertidal and supralittoral zones of Korean tidal flats increases the known biodiversity of these ecosystems. This finding highlights the importance of continued research on marine invertebrates in Korean tidal flats.

Further investigations are needed to clarify the distribution, ecology and population connectivity of *O. reevesii* not only in Korea, but also in East Asia. Expanded surveys targeting additional saltmarsh habitats along the western and southern coasts of the Peninsula may reveal a broader distribution than currently understood. Applying additional molecular markers and increasing sampling coverage will also help to elucidate potential genetic exchange between Korean and Chinese populations. Likewise, detailed anatomical studies would complement the external morphological and molecular data presented here. Overall, continued integrative taxonomic work will be essential for advancing our understanding of onchidiid diversity on the Korean Peninsula and East Asia. Such integrative taxonomic efforts go beyond documenting a single species and underscore the need for increased taxonomic attention to onchidiid slugs and other cryptic intertidal gastropods in East Asia coastal ecosystems.

## Supplementary Material

XML Treatment for Onchidium reevesii

## Figures and Tables

**Table 1.** Specimen information and GenBank accession numbers for COI and 16S rRNA sequences of *Onchidium reevesii* generated in this study. COI sequencing failed for specimen JD2.

Species	Locality	Sample ID	GenBank COI	GenBank 16S
*O. reevesii*	Suncheon, Korea	SC1	PX715933	PX715879
		SC2	PX715934	PX715880
		SC3	PX715935	PX715881
		SC4	PX715936	PX715882
	Muan, Korea	MA1	PX715937	PX715883
		MA2	PX715938	PX715884
		MA3	PX715939	PX715885
	Jindo, Korea	JD1	PX715940	PX715886
		JD2	-	PX715887

**Table 2.** The top five results for each of the NCBI BLAST searches, conducted on the COI and 16S regions of *Onchidium reevesii*, are presented in descending order of percentage similarity.

COI			16S		
BLAST search	Accession No. (bp)	Percentage Similarity	BLAST search	Accession No. (bp)	Percentage Similarity
*O. reevesii*	GU166527 (655)	99.83%	*O. reevesii*	GQ985205 (448)	99.55%
*O. reevesii*	GU166515 (655)	99.66%	*O. reevesii*	JQ625505 (407)	99.51%
*O. reevesii*	GU166528 (655)	99.66%	*O. reevesii*	GQ985211 (448)	99.33%
*O. reevesii*	JQ625521 (633)	99.64%	*O. reevesii*	GQ985233 (448)	99.33%
*O. reevesii*	JQ625543 (633)	99.64%	*O. reevesii*	GQ985243 (448)	99.33%
